# An Enhanced Transportation System for People of Determination

**DOI:** 10.3390/s24196411

**Published:** 2024-10-03

**Authors:** Uma Perumal, Fathe Jeribi, Mohammed Hameed Alhameed

**Affiliations:** College of Engineering and Computer Science, Jazan University, Jazan 45142, Saudi Arabia; uperumal@jazanu.edu.sa (U.P.); malhameed@jazanu.edu.sa (M.H.A.)

**Keywords:** bus identification, mean cross-covariance spectral subtraction (MCC-SS), people of determination, bus route identification, recurrent neural network (RNN), radio frequency identification (RFID), bus bay detection and transport guidance system

## Abstract

Visually Impaired Persons (VIPs) have difficulty in recognizing vehicles used for navigation. Additionally, they may not be able to identify the bus to their desired destination. However, the bus bay in which the designated bus stops has not been analyzed in the existing literature. Thus, a guidance system for VIPs that identifies the correct bus for transportation is presented in this paper. Initially, speech data indicating the VIP’s destination are pre-processed and converted to text. Next, utilizing the Arctan Gradient-activated Recurrent Neural Network (ArcGRNN) model, the number of bays at the location is detected with the help of a Global Positioning System (GPS), input text, and bay location details. Then, the optimal bay is chosen from the detected bays by utilizing the Experienced Perturbed Bacteria Foraging Triangular Optimization Algorithm (EPBFTOA), and an image of the selected bay is captured and pre-processed. Next, the bus is identified utilizing a You Only Look Once (YOLO) series model. Utilizing the Sub-pixel Shuffling Convoluted Encoder–ArcGRNN Decoder (SSCEAD) framework, the text is detected and segmented for the buses identified in the image. From the segmented output, the text is extracted, based on the destination and route of the bus. Finally, regarding the similarity value with respect to the VIP’s destination, a decision is made utilizing the Multi-characteristic Non-linear S-Curve-Fuzzy Rule (MNC-FR). This decision informs the bus conductor about the VIP, such that the bus can be stopped appropriately to pick them up. During testing, the proposed system selected the optimal bay in 247,891 ms, which led to deciding the bus stop for the VIP with a fuzzification time of 34,197 ms. Thus, the proposed model exhibits superior performance over those utilized in prevailing works.

## 1. Introduction

The World Health Organization has stated that there are approximately 290 million VIPs worldwide. Self-navigation is a major issue for these individuals in society [1]. VIPs depend on others to accomplish daily tasks, due to their inability to recognize objects, and have difficulty in locating transport services and bus stations [2]. Primarily, research has focused on detecting obstacles to enable safer movement of VIPs. To inform VIPs about the type of obstacle, its distance from the person, and its position, these objects can be detected using deep learning techniques [3].

Through utilizing a transfer learning technique, various objects can be detected in real time [4]. However, to ensure the safety of VIPs, it is necessary to detect moving objects near them. Thus, to alert users, a voice-assisted smart stick has been designed using Radio Frequency Identification (RFID) technology and ultrasonic sensors [5]. Utilizing OpenStreetMap and the General Transit Feed Specification (GTFS), a multi-modal route planning system has been developed to guide VIPs [6]. After planning routes, the safer navigation of VIPs is ensured through the utilization of a Web-Based Application (WBA). The object information is forwarded to the MobileNet architecture by the WBA camera. Regarding objects, MobileNet produces audio messages via a speech module [7]. In this way, the user can recognize obstacles, including vehicles, shopping stores, and traffic signals, using a deep attention network [8].

To date, various wearable devices based on GPS have been developed to facilitate access to public transport [9,10]. VIPs can be informed about the current location and arrival of buses through an Application Interface (API) and learned database of the device [11]. Generally, bus detection systems are constructed with microcontrollers, RF modules, sensors, and Bluetooth [12]. Utilizing the BlindMobi app, bus travel in urban centers is made easier for VIPs [13]. A VIP at a bus station can be recognized by the corresponding bus driver via the user’s RFID tag [14]. The data from such a tag are forwarded to an RFID reader and alerts the bus driver about the person’s destination via voice messages [15]. However, no study in the existing literature has concentrated on guiding VIPs at a bus station which contains multiple bus lines. Thus, this study proposes an optimal bay selection method using the EPBFTOA approach, enabling VIPs to better access bus transport.

### Problem Statement

The problems noticed in prevailing works are described below:

- A bus station containing more than one bus line is not considered in most of the prevailing works, thus causing difficulty for VIPs in recognizing the relevant bus line.
- The route information accessed from a single source is inefficient for VIPs who are making journeys.
- The VIP still needs human guidance to identify bus routes and bus numbers, or they need to enquire of bus coordinators.
- When there is a larger queue of buses at the bus station, it is difficult for VIPs to identify the correct bus, especially when the desired bus is farther away.
- The conversion of the VIP’s voice data can be affected by the presence of background noise, resulting in the wrong information being attained.
- In some existing works, computer vision has been used to capture the user’s surroundings for identification of buses. However, this approach is inefficient when nearby buses are not the appropriate transport for VIPs.
- Main goal: The main goal of the proposed study is to detect and select the optimal bay, as per the visually impaired person’s query, in order to promote the accessibility of bus transport. Through selection of the optimal bay, the difficulties and obstacles faced by the VIP regarding the determination of the location of the desired bus in the bus station are mitigated. Thus, based on the recognition of the optimal bay, bus transportation becomes more efficient for the visually impaired. The major contributions or objectives of the proposed transportation system for the visually challenged are further mentioned.

The proposed work’s main objectives are as follows:

- The optimal bay among the detected bus bays is selected using the EPBFTOA method, in order to recognize the appropriate bus line in the bus station.
- To access the correct bus, information from multiple sources is obtained, including the GPS location, voice data, bus image, and bus route.
- To minimize the need for human guidance, the VIP’s speech data are acquired using an Internet of Things (IoT) application via a mobile device to retrieve the details of the desired bus.
- The VIPs face difficulty in reaching the correct bus among the large queue of buses. Thus, an RFID sensor is utilized to inform the respective bus drivers who should carry the VIP.
- To retrieve correct information from the VIP, their voice data are pre-processed with the Mean Cross-Covariance Spectral Subtraction (MCC-SS) approach in order to remove background noise.
- Utilizing the SSCEAD method, the text is segmented from the bus, and the Levenshtein Distance (LD) measure is used to determine the route similarity.

The remainder of this paper is arranged as follows: The related works of this paper are discussed in Section 2. The proposed method is described in Section 3, and its performance is examined in Section 4. Finally, Section 5 concludes this paper with future scope.

## 2. Literature Survey

Authors [16] proposed a technique named ‘My Vision’, which enables VIPs to identify bus route numbers. Through the Lucas–Kanade tracker of My Vision images, the arriving bus is captured. Next, the bus board area is acquired utilizing the Random Forest (RF) technique. Moreover, the route number is extracted utilizing the pattern-matching approach. In addition, the detection rate of the proposed method was enhanced, when compared to traditional techniques. However, the relationships among features could not be evaluated by RF, potentially resulting in the inaccurate detection of bus route numbers.

Authors [17] recommended a navigation system for VIPs in a multi-obstacle scenario. Through a query processor, the person’s query was accessed. The YOLO version 3 (YOLOv3) model is used to detect various obstacles, and the optimal path is selected utilizing the Environment-aware Bald Eagle Search algorithm. The performance was improved regarding latency and detection accuracy; however, the Actor–critic algorithm utilized for navigation decisions led to trade-offs, and the decisions made were not reliable.

Authors [18] established a wearable device for the safe traveling of VIPs. The bus is detected utilizing the YOLOv3 technique, and the bus board is segmented with a transfer learning technique. Next, the bus number obtained is transformed into a voice. The bus board detection accuracy was improved over prevailing techniques; however, smaller objects could not be recognized by the anchor box of YOLOv3, thus limiting the efficiency.

Authors [19] explored a device to assist in the navigation of blind persons. The user is informed to make a decision regarding the safer path through the use of a speech generation device. The Robot Operating System minimized the occurrence of distractions. Next, through a Fuzzy logic system, the safe directions are issued to the VIP. When compared to other approaches, the recommended device attained a lower collision rate; however, the Fuzzy rules were simply developed based on assumptions, and thus, an accurate decision was not produced.

Authors [20] suggested a smart glass system for the independent movement of blind persons at night-time. To detect the object accurately using the U2-Net model, the path image is pre-processed and represented as a tactile graph. Moreover, the text in the image is converted to speech. When compared to other networks, this model detected objects with higher accuracy and precision; however, the Tesseract model could not effectively extract text from low-quality and poorly lit images.

Authors [21] proposed a Commute Booster (CB) mobile application for the navigation support of blind persons. From the GTFS dataset, way-finding footage is obtained. Next, the Optimal Character Recognition (OCR) system is utilized to enable VIPs to identify the relevant path. When compared to prevailing techniques, the performance of the presented approach was improved, regarding precision, accuracy, and f1-score; however, the OCR could not process image data in different formats.

Authors [22] presented a lightweight bus detection approach for VIPs utilizing the YOLO network. To detect buses in real-time, the structure of YOLO is modified with a slim scale detection module. This model detected the bus with higher accuracy and precision; however, the bus detection performance was non-optimal, as the YOLO model was processed with fewer parameters.

Authors [23] proposed a framework named ‘Vision Navigator’ for the blind and VIPs utilizing a Recurrent Neural Network (RNN). To detect the presence of an object in the path, a stick utilizing the single-shot mechanism is created. Moreover, obstacles within shorter distances are detected via sensor-equipped lightweight shoes. The developed framework could detect obstacles with a high accuracy rate; however, the RNN did not learn complex data patterns due to the gradient vanishing problem, which degraded the framework’s efficiency.

Authors [24] established a mobile application for VIPs to identify bus stops. The bus stop signs were detected through a mobile camera with the All-Aboard Application (AAA) utilizing a neural network. The VIP can assess a bus stop within a distance of 30 to 50 m via AAA. Regarding the distance between actual bus stop locations and the indicated location, the performance was analyzed. However, for accurate detection, the application needed a large number of labeled data and high-quality images.

Authors [25] explored a wearable device to assist VIPs in navigation. The device was developed for use with a camera embedded on a smartphone or eyeglasses. Utilizing a Convolutional Neural Network (CNN), the object detection system was developed and deployed in the smart phone. The performance was evaluated regarding its efficiency and safety. However, its capacity for object detection was limited by the usage of a CNN, as it could not efficiently learn sequential data.

The related works are comparatively summarized in Table 1.

## 3. Proposed Methodology

**Figure 1:**
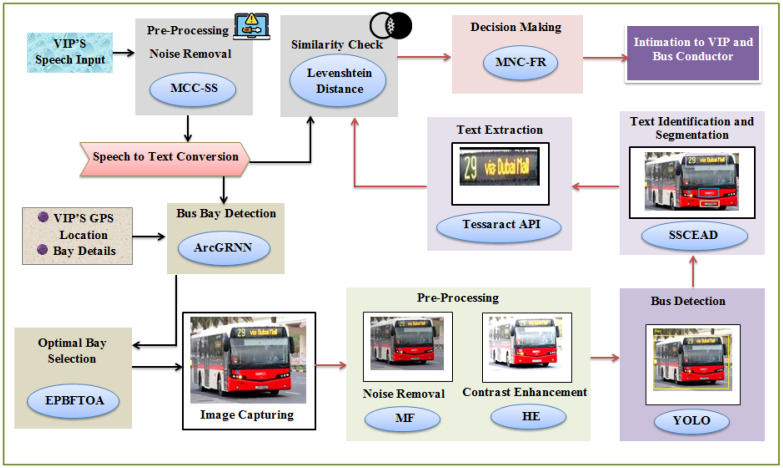
Framework of the proposed model.

Using the MNC-FR and EPBFTOA methods, the proposed model comprises a transportation system that better enables VIPs to travel by bus. First, the bus bays are detected, and an image is captured from the optimal bay. Then, the text on buses is extracted from the captured image, in order to determine their destinations. Finally, a decision is made, and the VIP and bus conductor are consequently informed. Figure 1 depicts the proposed model’s architecture.

### 3.1. Speech Input

Initially, through utilizing an IoT application, the destination details of the VIP are gathered in the form of speech from their mobile device. Let the input speech $\left(A\right)$ be signified as

(1) $$A=\left[A^{1},A^{2},A^{3},A^{4},\ldots ,A^{q}\right],$$

where the number of input words is denoted as $\left(q\right)$. The input $\left(A\right)$ is then pre-processed as follows.

### 3.2. Speech Pre-Processing

Next, employing the MCC-SS method, the voice data $\left(A\right)$ are pre-processed to remove the background noise. To obtain a clean speech signal, Spectral Subtraction (SS) is used, which subtracts the noise spectrum present in the original audio. Nevertheless, fixed parameters that do not adapt to the noise level in the input are utilized as a subtraction factor. To mitigate this issue, the Mean Cross-Covariance (MCC), which analyzes each signal of the input, is employed to determine the subtraction parameter. The MCC-SS process is described as follows:

The cross-covariance $\left(\alpha \right)$ between signals $\left(E,F\right)$ present in the speech input $\left(A\right)$ is computed as

(2) $$\alpha =\sum_{q}^{A}\left(E-\hat{E}\right)\times \left(F-\hat{F}\right),$$

where the mean value of $\left(E,F\right)$ is denoted as $\left(\hat{E},\hat{F}\right)$. The value $\left(\alpha \right)$, which is the obtained subtraction factor, is utilized to remove the input’s background noise. It is calculated as

(3) $$A^{\prime }=A-\alpha ,$$

where the pre-processed audio is signified as $\left(A^{\prime }\right)$.

### 3.3. Speech-to-Text Conversion

The pre-processed speech $\left(A^{\prime }\right)$ is additionally converted into the text format, in order to obtain the VIP’s destination and detect the bus bay in the road traffic. The text format is also employed to check the similarity between the VIP’s bus destination and bus route. Let the text converted from speech $\left(A^{\prime }\right)$ be denoted as $\left(T\right)$.

### 3.4. Bus Bay Detection

Utilizing ArcGRNN, the number of bus bays for the VIP is detected. The inputs employed for detecting the bus bays are as follows:

- The VIP’s destination $\left(T\right)$, in the form of text.
- The VIP’s location $\left(\beta \right)$, based on GPS.
- Bay details $\left(d\right)$ concerning numerous locations, obtained from the cloud database.

**Figure 2:**
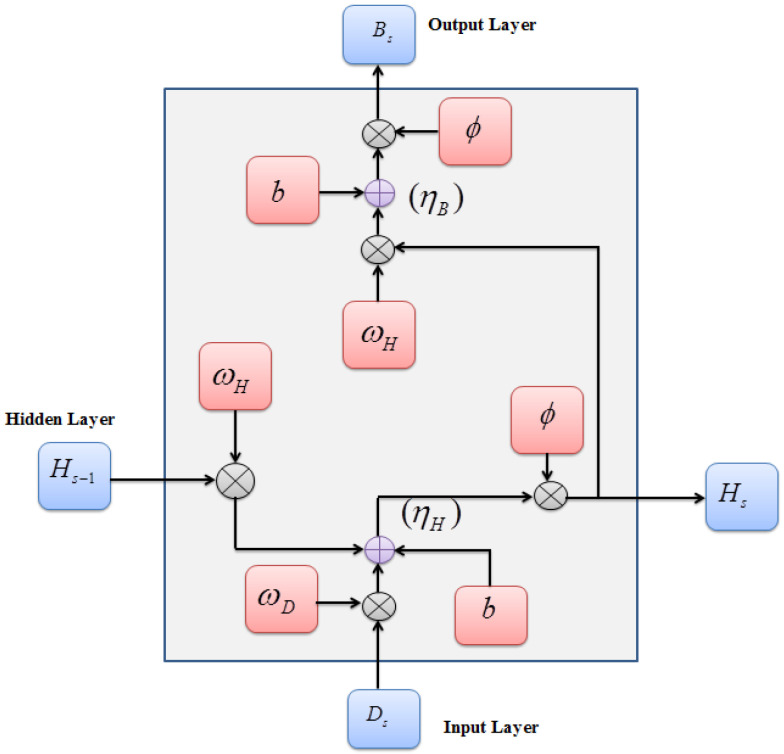
Architecture of ArcGRNN.

An RNN, which can handle inputs of varying lengths and provides an output ranging from single- to multi-class classification, was utilized for bus bay detection. Even though the RNN can process memory and binary data, the vanishing and exploding gradient problems may occur during the backpropagation process. Therefore, to prevent this issue, the Arctan Gradient Activation Function (Arc-GAF), which automatically enlarges small inputs and blocks large inputs, was used as an activation function in the RNN. Figure 2 illustrates the framework of ArcGRNN.

The ArcGRNN process is described as follows:

- Input Layer

The inputs required for the detection of bus bays are combined as follows:

(4) $$D_{s}=T+\beta +d,$$

where the VIP’s location is denoted as $\left(\beta \right)$, which is employed to identify the number of bays present in the specified location. From the cloud database, the bay details $\left(d\right)$ of various locations are obtained. The input $\left(D_{s}\right)$ concerning time $\left(s\right)$ is passed to the hidden layer for further processing.

- Hidden Layer

The hidden layer collects the information from the previous output and computes the input along with the activation function. The designed hidden layer’s input $\left[\eta_{H}\right]$ is given by

(5) $$\eta_{H}=\left[\left(D_{s}\times \omega_{D}\right)+\left(H_{s-1}\times \omega_{H}\right)\right]+b,$$

where the hidden layer’s previous output concerning time $\left(s-1\right)$ is signified as $\left(H_{s-1}\right)$, $\left(\omega_{D},\omega_{H}\right)$ are the weights of the input and hidden layers, respectively, and the bias value is denoted as $\left(b\right)$.

Next, utilizing Arc-GAF, the computed value of the hidden state is activated. The Arc-GAF takes in the required input to avoid the gradient-related issues of RNNs. The Arc-GAF activation function $\left(\phi \right)$ is given by

(6) $$\phi =\frac{g\times h}{1+{\left[h*\left(D_{s}\right)\right]}^{2}}-1,$$

where the parameters of the input $\left(D_{s}\right)$ are denoted as $\left(g,h\right)$. Then, the hidden layer’s output $\left(H_{s}\right)$ is calculated as follows:

(7) $$H_{s}=\eta_{H}\left(H_{s}\right)*\phi .$$

The output $\left(H_{s}\right)$ is further passed on to the output layer to obtain the final classification output.

- Output layer

Regarding $\left(H_{s}\right)$ and the weight $\left(\omega_{H}\right)$ of the hidden layer, the output layer $\left[\eta_{B}\right]$ is computed as follows:

(8) $$\eta_{B}=\left(H_{s}\times \omega_{H}\right)+b.$$

Utilizing $\left(\phi \right)$, the value $\left[\eta_{B}\right]$ is activated to give the final output $\left(B_{s}\right)$, as follows:

(9) $$B_{s}=\left[\eta_{B}\right]*\phi ,$$

(10) $$B_{s}=\left\{B_{s}^{1},B_{s}^{2},B_{s}^{3},\ldots ,B_{s}^{k-1},B_{s}^{k}\right\},$$

where $\left(B_{s}\right)$ are the detected bays for the respective VIP, and the number of detected bays is denoted as $\left(k\right)$. The pseudocode for the ArcGRNN model is given below as Algorithm 1:
**Algorithm 1** Pseudocode for ArcGRNN**Input:** VIP’s destination $\left(T\right)$, location $\left(\beta \right)$, bay details $\left(d\right)$ **Output:** Detected bay $\left(B_{s}\right)$ **Begin**   **Initialize** parameters $\left(\omega_{D},\omega_{H}\right)$, $\left(b\right)$   **For** $\left(s\right)$     **While** input $D_{s}=T+\beta +d$        **Calculate** Hidden layer input          $\eta_{H}=\left[\left(D_{s}\times \omega_{D}\right)+\left(H_{s-1}\times \omega_{H}\right)\right]+b$        **Evaluate** activation function $\left(\phi \right)$          $\phi =\frac{g\times h}{1+{\left[h*\left(D_{s}\right)\right]}^{2}}-1$        **Hidden** layer output $H_{s}=\eta_{H}\left(H_{s}\right)*\phi$        **Vectorize** output layer’s input          $\eta_{B}=\left(H_{s}\times \omega_{H}\right)+b$        **Find** final output $B_{s}=\left[\eta_{B}\right]*\phi$     **End** while   **End** for   **Obtain** detected bays $\left(B_{s}\right)$ **End**

Next, optimal bays are chosen from the detected bus bays $\left(B_{s}\right)$, as described below.

### 3.5. Optimal Bay Selection

Subsequently, utilizing EPBFTOA, the optimal bay is chosen from the detected bays $\left(B_{s}\right)$. The optimal bay is the bay line of the bus station, which should be utilized by the VIP to catch the desired bus. In this context, for optimal bay selection, the Bacteria Foraging Optimization Algorithm (BFOA), which mimics the foraging strategy of bacteria to select the best value, is utilized. Nevertheless, BFOA may suffer from premature convergence, thereby affecting the outcome of the optimizer. To solve this issue, the Experienced Perturbed Adaptive Search (EPAS) mechanism with Triangular Mutation is utilized in BFOA, which computes health and reproduction parameters for the bacteria to overcome the abovementioned premature convergence problem. 

The EPBFTOA is explained below:

- Initialization

The detected bays $\left(B_{s}\right)$, which are considered as bacteria, are the search agents. There are $\left(k\right)$ number of bacteria to search the nutrients (number of bays). The $\left(f\mathrm{th}\right)$ bacterium’s position is initialized as follows:

(11) $$B_{s}^{f}\left(\delta ,Z,\mu \right)=\left(B_{s}^{1},B_{s}^{2},B_{s}^{3},\ldots ,B_{s}^{k}\right),$$

where $\left(B_{s}^{f}\right)$ is the bacteria’s position concerning the chemotaxis $\left(\delta \right)$, $\left(Z\right)$ is the reproduction value, and $\left(\mu \right)$ is the elimination dispersal step parameters.

- Fitness

Concerning the minimum distance $\left(\lambda \right)$ between bacteria and nutrients, the fitness value $\left(\theta \right)$ that is employed to obtain the optimal bay is computed. The fitness function $\left(\theta \right)$ is defined as

(12) $$\theta =\min\left(\lambda \right).$$

The EPBFTOA encompasses four foraging strategies; namely, chemotaxis, swarming, reproduction, and elimination and dispersal.

- Chemotaxis

In this strategy, a bacterium chooses a favorable environment by swimming and tumbling. The bacterium’s movement concerning the swimming step $\left(R\right)$ and swimming direction $\left(v\right)$ is given by

(13) $$B_{s}^{f}\left(\delta +1,Z,\mu \right)=B_{s}^{f}\left(\delta ,Z,\mu \right)+\frac{R\left(f\right)\times l}{\sqrt{v^{T}\left(f\right)\times v\left(f\right)}}*v\left(f\right),$$

where the number of swimming steps taken by the $\left(f\mathrm{th}\right)$ bacteria is signified as $\left(l\right)$.

- Swarming

The bacteria’s swarming behavior $C\left(B_{s}^{f}\right)$ after chemotaxis is centered on the attraction $\left(a\right)$ and repulsion $\left(r\right)$ of the bacteria, defined by

(14) $$\begin{array}{l} C\left(B_{s}^{f}\right)=\sum_{f=1}^{k}\left\{-x^{a}\times \exp\left[y^{a}\sum {\left(B_{s}^{f}\left(\delta ,Z,\mu \right)-B_{s}^{f}\left(\delta +1,Z,\mu \right)\right)}^{2}\right]\;\;\right\}\;\\ \;\;\;\;\;\;\;\;\;\;\;\;\;\;\;\;\;\;+\;\;\;\sum_{f=1}^{k}\left\{x^{r}\times \exp\left[y^{r}\sum {\left(B_{s}^{f}\left(\delta ,Z,\mu \right)-B_{s}^{f}\left(\delta +1,Z,\mu \right)\right)}^{2}\right]\;\;\right\}\; \end{array},$$

where the depth and width regarding bacterial attraction are denoted as $\left(x^{a},y^{a}\right)$, and $\left(x^{r},y^{r}\right)$ are the depth and width regarding bacterial repulsion.

The bacteria’s new position ${\hat{B}}_{s}^{f}\left(\delta +1,Z,\mu \right)$ regarding swarming is given by

(15) $${\hat{B}}_{s}^{f}\left(\delta +1,Z,\mu \right)=B_{s}^{f}\left(\delta +1,Z,\mu \right)+C\left(B_{s}^{f}\right).$$

- Reproduction

In this strategy, healthier bacteria are located to attain better optimization outcomes. Initially, centered on EPAS, the healthier bacteria $\left({\hat{B}}_{best}\right)$ are computed. Regarding the fitness function, the best bacteria are identified by the EPAS as follows:

(16) $${\hat{B}}_{best}={\hat{B}}_{mean}+\left(\tau \times {\hat{B}}_{dev}\right),$$

(17) $${\hat{B}}_{mean}=\frac{\left({\hat{B}}_{s}^{f}+\theta \right)}{2},$$

(18) $${\hat{B}}_{dev}={\hat{B}}_{s}^{f}-\theta ,$$

where the mean and deviation of the healthier bacteria with random values $\left(\tau \right)$ are denoted as $\left({\hat{B}}_{mean},{\hat{B}}_{dev}\right)$. Next, the reproduction value ${\hat{B}}_{s}^{f}\left(\delta +1,Z+1,\mu \right)$ is obtained by Triangular Mutation, which gives the output concerning the best $\left({\hat{B}}_{best}\right)$, the worst $\left({\hat{B}}_{wor}\right)$, and better $\left({\hat{B}}_{bet}\right)$ bacteria, as follows:

(19) $${\hat{B}}_{s}^{f}\left(\delta +1,Z+1,\mu \right)={\hat{B}}_{best}+p_{1}\left({\hat{B}}_{best}-{\hat{B}}_{bet}\right)+Z,$$

(20) $$Z=p_{2}\left({\hat{B}}_{best}-{\hat{B}}_{wor}\right)+p_{3}\left({\hat{B}}_{bet}-{\hat{B}}_{wor}\right),$$

(21) $${\hat{B}}_{wor}={\hat{B}}_{s}^{f}-{\hat{B}}_{best},$$

(22) $${\hat{B}}_{bet}={\hat{B}}_{s}^{f}\approx {\hat{B}}_{best},$$

where the mutation factors of the bacteria are signified as $\left(p_{1},p_{2},p_{3}\right)$.

- Elimination and Dispersal

After reproduction, the bacteria with lower probability dies and provide their optimal solution, with respect to their random dispersion $\left(\kappa \right)$ and probability $\left(P\right)$, as follows:

(23) $${\hat{B}}_{s}^{f}\left(\delta +1,Z+1,\mu +1\right)=\left\{\begin{array}{l} {\hat{B}}_{s}^{f}\left(\delta +1,Z+1,\mu \right)\;\;\;\;\forall \left(\kappa >P\right)\\ B^{*}\;\;\;\;\forall \left(\kappa <P\right) \end{array}\right..$$

Thus, through using the EPBFTOA approach, the optimal bus bay value $\left(B^{*}\right)$ is obtained. The pseudocode for this optimizer is given below in Algorithm 2.
**Algorithm 2** Pseudocode for EPBFTOA**Input:** Detected Bay $\left(B_{s}\right)$ **Output:** Optimal Bus Bay $\left(B^{*}\right)$
**Begin**   **Initialize** bacteria population $\left(B_{s}^{f}\right)$, Iteration $\left(\int ,\int^{\max}\right)$, $\left(\kappa ,P\right)$     $B_{s}^{f}\left(\delta ,Z,\mu \right)=\left(B_{s}^{1},B_{s}^{2},B_{s}^{3},\ldots ,B_{s}^{k}\right)$   **Calculate** fitness $\theta =\min\left(\lambda \right)$   **While** $\left(\int \leq \int^{\max}\right)$     **For** $\left(\theta \right)$        **Move** bacteria by chemotaxis $B_{s}^{f}\left(\delta +1,Z,\mu \right)$        **Swarm** bacteria          ${\hat{B}}_{s}^{f}\left(\delta +1,Z,\mu \right)=B_{s}^{f}\left(\delta +1,Z,\mu \right)+C\left(B_{s}^{f}\right)$        **Reproduce** bacteria          ${\hat{B}}_{s}^{f}\left(\delta +1,Z+1,\mu \right)={\hat{B}}_{best}+p_{1}\left({\hat{B}}_{best}-{\hat{B}}_{bet}\right)+Z$        **Eliminate** and disperse ${\hat{B}}_{s}^{f}\left(\delta +1,Z+1,\mu +1\right)$          **If**
$\left(\kappa <P\right)$            **Optimal** bus bay $\left(B^{*}\right)$        **Else**          **Original** position ${\hat{B}}_{s}^{f}$        **End** if     **End** for   **End** while   **Return** optimal bus bay $\left(B^{*}\right)$ **End**

After the selection of the optimal bay $\left(B^{*}\right)$, the image is captured using the mobile device of the VIP. Then, the image is pre-processed as detailed below.

### 3.6. Image Capturing and Preprocessing

From the optimal bay $\left(B^{*}\right)$, bus images are captured. These are then pre-processed for noise removal and contrast enhancement. Let $\left(j\right)$ the number of images $\left(G\right)$ captured via the mobile device of VIPs be represented as

(24) $$G=\left[G^{1},G^{2},G^{3},\ldots ,G^{j}\right].$$

In this way, unwanted artifacts are removed and the quality of the image is enhanced to make further processing more effective. The pre-processing of $\left(G\right)$ is described below.

#### 3.6.1. Step 1: Noise Removal

Due to noises such as salt and pepper and speckle noise, the quality of the image is affected. Therefore, the Median Filter (MF)—which eliminates noisy pixels—is utilized for the removal of noise from the image $\left(G\right)$, which is calculated as follows:

(25) $$G^{\prime \prime }=G\left(t,u\right)\times \frac{\left(t+u\right)}{2},$$

where the coordinates of pixels in the image $\left(G\right)$ are denoted as $\left(t,u\right)$, and the noise-removed image, which is used for subsequent contrast enhancement, is denoted as $\left(G^{\prime \prime }\right)$.

#### 3.6.2. Step 2: Contrast Enhancement

Histogram Equalization (HE), which adjusts the contrast using the image’s histogram, is used to make the image clearer for further processing. The HE is elaborated as follows:

Regarding histogram values such as the pixel value $\left(c\right)$ and number of pixels $\left(\Pi \right)$ in the image $\left(G^{\prime \prime }\right)$, the probability of occurrence $\left(J\right)$ is given by

(26) $$J=\frac{{\left(\Pi \right)}_{c}}{\Pi }\;\;\;\;\forall \left(0\leq c\leq W\right),$$

where the number of pixels regarding $\left(c\right)$ is denoted as $\left(\Pi_{c}\right)$, and the total grayscale value of $\left(G^{\prime \prime }\right)$ is denoted as $\left(W\right)$. Then, the cumulative distribution function $\left(\psi \right)$ is computed as:

(27) $$\psi =\sum_{c}J\left(G^{\prime \prime }\right).$$

Finally, the image’s contrast $\left(G^{\prime \prime }\right)$ is improved as follows:

(28) $$G^{*}=\psi \times G^{\prime \prime }.$$

The contrast-enhanced image $\left(G^{*}\right)$ is the pre-processed image, and the bus present in this image is detected as explained below.

### 3.7. Bus Detection

Through employing a YOLO series model, buses are detected in the pre-processed image $\left(G^{*}\right)$. The YOLO model splits the images into grids and detects objects concerning the bounding boxes of the grids. The YOLO model’s process is explained in the following.

Initially, the image $\left(G^{*}\right)$ is split into a $\left(M\times M\right)$ grid pattern. These grids have a number $\left(e\right)$ of bounding boxes $\left(U\right)$, which are signified as:

(29) $$U=\langle U_{1},U_{2},U_{3},\ldots ,U_{e}\rangle .$$

Each bounding box, with height $\left(z\right)$, width $\left(w\right)$, and coordinates $\left(l,m\right)$ is denoted as:

(30) $$U\to U\left(z,w,l,m\right).$$

The bounding boxes might overlap each other, and therefore, the degree of overlap $\left(\varsigma \right)$ concerning bounding boxes $\left(U_{1},U_{2}\right)$ is computed as:

(31) $$\varsigma =\frac{U_{1}\cap U_{2}}{U_{1}\cup U_{2}}.$$

Finally, utilizing $\left(U\right)$ and $\left(\varsigma \right)$, the objects can be detected, which are given as:

(32) $$N=U\left(z,w,l,m\right)*\;\varsigma .$$

A bus detected in the image is symbolized as $\left(N\right)$ and from this, the text is identified and segmented for further analysis.

### 3.8. Text Identification and Segmentation

Next, for identification of the destination of the bus, the text present in the detected bus image $\left(N\right)$ is identified and segmented. For this purpose, Encoder–Decoder (ED) processing is carried out. In this process, a CNN, which analyzes each input accurately, is utilized for encoding. To enhance the learning ability of the CNN, the Sub-pixel Shuffling Convolution (SSC) strategy is used in the convolution layer, thus expanding the receptive field of the CNN. A Bidirectional Long Short-Term Memory (BiLSTM) model is typically used as a decoder in the ED process; however, text identification becomes difficult, as BiLSTM models have high complexity. Thus, to enhance the text identification and segmentation effect, ArcGRNN is used as the decoder in this study. The SSCEAD process is elaborated further in the following.

- Encoder

Utilizing the Sub-pixel Shuffling Convolution Encoder (SSCE), the text is first recognized in the image $\left(N\right)$. Initially, the pixels $\left(K\times L\right)$ of the input image are reshuffled in the convolutional layer of the CNN using SSC. Then, the encoder process is executed for the reshuffled image. The image $\left(\bar{N}\right)$ that is obtained using SSC, with an up-sampling factor $\left(i\right)$, is given as

(33) $$\bar{N}=\left[i\left(K\right)\times i\left(L\right)\right].$$

Then, regarding the image $\left(\bar{N}\right)$ and the Rectified Linear Unit (ReLU) activation function $\left(℘\right)$, the convolutional layer’s output $\left(Q\right)$ is calculated as

(34) $$Q=\left\{\;\left[\bar{N}\times \varpi_{Q}\right]+o\right\}*℘,$$

(35) $$℘=\max\left(\varepsilon^{1},\varepsilon^{2}\right),$$

where the coordinates of the image $\left(\bar{N}\right)$ are $\left(\varepsilon^{1},\varepsilon^{2}\right)$, the weight value is denoted as $\left(\varpi_{Q}\right)$, and the bias value of the image $\left(\bar{N}\right)$ is signified as $\left(o\right)$. The value $\left(Q\right)$ is max-pooled and fully connected to give the encoded output $\left(I\right)$:

(36) $$I=\left[\sum \left(\max\left(Q\right)\times \varpi_{I}\right)+o\right]*℘,$$

where the text-detected image is denoted as $\left(I\right)$. This image is then decoded as follows:

- Decoder

In order to determine the destination of the bus, the text-identified image $\left(I\right)$ is decoded. The ArcGRNN decoder methodology, which processes the input concerning the prior information, enlarges the input size and blocks the large input (note that the process of the ArcGRNN technique is explained in Section 3.4). The image $\left(I\right)$ is processed via the hidden and output layers, along with Arc-GAF. The text-segmented image $\left(S\right)$, which represents the bus destination, is the obtained output. Subsequently, the text is extracted from $\left(S\right)$ as detailed below.

### 3.9. Text Extraction

Next, regarding the bus destination image $\left(S\right)$, the bus route is identified utilizing the Tesseract API, which is an OCR model. In this model, the text is extracted automatically from $\left(S\right)$. Initially, the fixed pitch of the text present in the image is found. After that, the characters are split into words and are automatically recognized and extracted in text form for the fixed pitch. Let the extracted text be signified as $\left(n\right)$, which is the destination of the bus in text form. Therefore, with respect to the bus route details obtained from the cloud database, the bus route $\left(V\right)$ is identified from $\left(n\right)$. Subsequently, to make the decision command for transportation, the similarity between the VIP’s destination and the route and destination of the bus is checked.

### 3.10. Similarity Check

Next, the similarity analysis between the bus destination $\left(n\right)$, bus route $\left(V\right)$, and VIP’s destination $\left(T\right)$ is performed. For similarity analysis, the LD, which measures the similarity between two parameters, is used. First, the similarity between $\left(n\right)$ and $\left(T\right)$ is checked as follows. Let the length of $\left(n\right)$ be $\left(\gamma \right)$ and length of $\left(T\right)$ be $\left(ƛ\right)$. Then, the similarity $\left(O\right)$ between $\left(n\right)$ and $\left(T\right)$ is calculated as

(37) $$O\left(n,T\right)=\left\{\begin{array}{ll} 0 & if\left(ƛ=0\right)\\ 0 & if\left(\gamma =0\right)\\ 1 & if\left(\gamma =ƛ\right)\\ \min\left\{\begin{array}{l} O\left(\gamma -1,ƛ\right)+1\\ O\left(\gamma ,ƛ-1\right)+1\\ O\left(\gamma -1,ƛ-1\right)+1 \end{array}\right. & \left(otherwise\right) \end{array}\right..$$

Equation (37) states that the similarity value $\left(O\right)$ becomes higher when the length of the destinations is similar. In addition, if there are changes in lengths, then the minimum conditions are followed to obtain the similarity score. If $\left(O\right)$ is high, this similarity value is used for decision-making. Otherwise, the similarity $\left(O^{*}\right)$ between $\left(V\right)$ and $\left(T\right)$ is checked further:

(38) $$O\left(n,T\right)=\left\{\begin{array}{ll} 0 & if\left(ƛ=0\right)\\ 0 & if\left(\sigma =0\right)\\ 1 & if\left(\sigma =ƛ\right)\\ \min\left\{\begin{array}{l} O\left(\sigma -1,ƛ\right)+1\\ O\left(\sigma ,ƛ-1\right)+1\\ O\left(\sigma -1,ƛ-1\right)+1 \end{array}\right. & \left(otherwise\right) \end{array}\right..$$

Here, the length of the words present in the bus route is denoted as $\left(\sigma \right)$. The similarity value is finally given for decision-making, as detailed below.

### 3.11. Decision-Making

Finally, regarding the similarity score between the VIP destination and the bus destination $\left(O\right)$, as well as the similarity score between the bus route and the VIP destination $\left(O^{*}\right)$, the decision is made to inform both the VIP and the bus conductor. In this context, the Fuzzy Rule (FR), which is used to analyze the input parameters efficiently, is utilized for decision-making. However, a probability measure that might make the output value zero is utilized in the Fuzzy logic approach. Therefore, a solution in decision-making may not be attained with the FR. Therefore, to mitigate this issue, a Multi-characteristic Index (MI) that gives non-zero output in Fuzzy logic is employed to compute the Fuzzy relationship. In addition, the Non-linear S-Curve (NC) membership function, which explains the certainty of the Fuzzy inputs, is used rather than the Fuzzy membership function. The MNC-FR approach is described further below.

- Rule

Initially, based on the if–then condition, the rules $\left(ℜ\right)$ are set for the decision-making as follows:

(39) $$ℜ=\left\{\begin{array}{l} if\;O\;is\;high\;then\;\Omega \\ if\;O\;is\;low\;then\;\Theta \\ if\;O^{*}is\;high\;then\;\Omega \\ if\;O^{*}\;is\;high\;then\;\Theta \end{array}\right.,$$

where the decision-making factor is denoted as $\left(\Omega \right)$ and the non-decision factor is signified as $\left(\Theta \right)$. The rule states that the decision to inform the VIP and the bus conductor is made when the similarity scores are high; otherwise, no decision is made. The image capturing process continues until a high similarity is obtained.

- Membership Function

The NC membership function $\left(X\right)$ is used to change the output value automatically, concerning the input data. To find the degree of relationship to the input parameter, the NC membership function is used. Therefore, $\left(X\right)$ is calculated as

(40) $$X=\left\{\begin{array}{ll} 1 & \forall \left(ℵ<\bar{ℵ}\right)\\ 0.999 & \forall \left(ℵ=\bar{ℵ}\right)\\ \hbar /\left(1+\left(\upsilon *e^{ℵ}\right)\right) & \forall \left(\bar{ℵ}<ℵ<{ℵ}^{\prime \prime }\right)\\ 0.0001 & \forall \left(ℵ={ℵ}^{\prime \prime }\right)\\ 0 & \forall \left(ℵ>{ℵ}^{\prime \prime }\right) \end{array}\right.,$$

where the scaling parameters of the input are denoted as $\left(ℵ,\bar{ℵ},{ℵ}^{\prime \prime }\right)$, and the constant values are signified as $\left(\hbar ,\upsilon \right)$. According to Equation (40), the membership function values are obtained in the range of 0 to 1 regarding the scaling parameter conditions.

- Fuzzification

In this step, the input data are converted to Fuzzy data $\left(\vartheta \right)$, such that the FR methodology can be used to enable further processing. Let the inputs $\left(O\right)$ and $\left(O^{*}\right)$ be combined and represented by $\left(\Xi \right)$. The fuzzification is then performed as follows:

(41) Ξ→ϑ.

- Fuzzy Relationship

To make the final decision, the relationship between the Fuzzy data $\left(\vartheta \right)$ is determined. To calculate the Fuzzy relationship, the MI that gives the optimal decision accurately is used, which is given as

(42) $$Y=\frac{\left(\sum \vartheta *X\left(ℜ\right)\right)}{X},$$

where the final decision obtained using the MNC-FR methodology is denoted as $\left(Y\right)$. These data are then converted back into crisp data.

- Defuzzification

For the purpose of intimation, the data $\left(Y\right)$ are converted into measurement data $\left(Y^{*}\right)$ as follows:

(43) $$Y^{*}=\left(\sum Y\times X\right)/\sum X.$$

Hence, the decision is made from $\left(Y^{*}\right)$ to guide the VIP onto the designated bus through giving information to the VIP through an RFID signal. Simultaneously, it also informs the bus conductor when to stop the bus, taking the VIP’s current location into consideration. Therefore, an enhanced transportation system for the VIP to travel by bus can be designed through the utilization of the proposed methodology. In Section 4, the performance analysis of the proposed approach is detailed.

## 4. Results and Discussions

The performance of the proposed model was evaluated by comparing it with existing approaches. The proposed work was implemented using the PYTHON 3.11 software in order to analyze the performance.

### 4.1. Dataset Description

Bus identification was performed utilizing the data in a public bus transport dataset regarding Dubai Bus Transportation. The dataset contains the route ID, trip ID, stop ID, bus stop name, and number of boardings data. By utilizing the Geospatial Bus Route Analysis (GBRA) dataset, the bus route similarity based on the speech requirements of VIPs was determined. In the GBRA dataset, the Bus Route Transit (BRT) route details, non-BRT route details, route type, route description, and bus stop names are present. Moreover, the proposed model was validated utilizing the Microsoft Common Objects in Context (MS-COCO) dataset. This dataset consists of 328,000 images in more than 80 object categories. From all these datasets, the data were split with a ratio of 70:20:10 for training, testing, and validation of the proposed model, respectively.

Table 2 displays the obtained results for the captured bus images, noise-removed images, contrast-enhanced images, and text-segmented images using the proposed bus identification method.

### 4.2. Performance Assessment

The proposed system’s performance was examined with respect to optimal bay selection, decision-making, text detection, and similarity between the bus route and voice data of the VIPs. Through comparing the proposed method with state-of-the-art algorithms including BFOA, Manta Ray Foraging Optimization (MRFO), African Vultures Optimization Algorithm (AVOA), and Bald Eagle Search Optimization (BESO), the time taken to select the optimal bay and fitness over iteration were evaluated.

Table 3 analyzes the time and fitness metrics of the proposed technique. In order to select the optimal bay, the proposed EPBFTOA took 247,891 ms, less than that of the prevailing techniques; in particular, for optimal bay selection, the BFOA, MRFO, AVOA, and BESO approaches took 389,124 ms, 596,757 ms, 804,628 ms, and 997,245 ms, respectively. The proposed algorithm selected the optimal bay within a minimum duration as the proposed algorithm compensates for the premature convergence problem in the selection process. When compared to the prevailing algorithms, the average fitness (9.068) attained by the proposed algorithm was lower, indicating the proposed EPBFTOA technique had enhanced performance in selecting the optimal bay.

**Figure 3:**
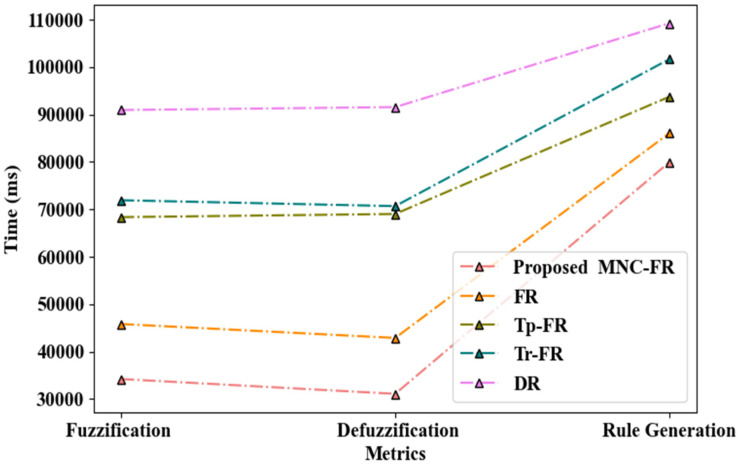
Graphical representation of the proposed decision generation approach.

Figure 3 represents the performance of decision-making to reach the relevant bus for the VIP using the proposed MNC-FR technique. It can be observed that the MNC-FR technique performed fuzzification within 34,197 ms and de-fuzzification in 31,073 ms, and thus, generated the decision rule within 79,932 ms. When compared to prevailing techniques such as the FR, Trapezoidal Fuzzy Rule (Tp-FR), Triangular Fuzzy Rule (Tr-FR), and Decision Rule (DR), the decision time of the proposed technique was shorter. The proposed MNC-FR takes less time to determine the relevant bus as the similarity between the bus route and voice data is analyzed before generating a decision.

**Figure 4:**
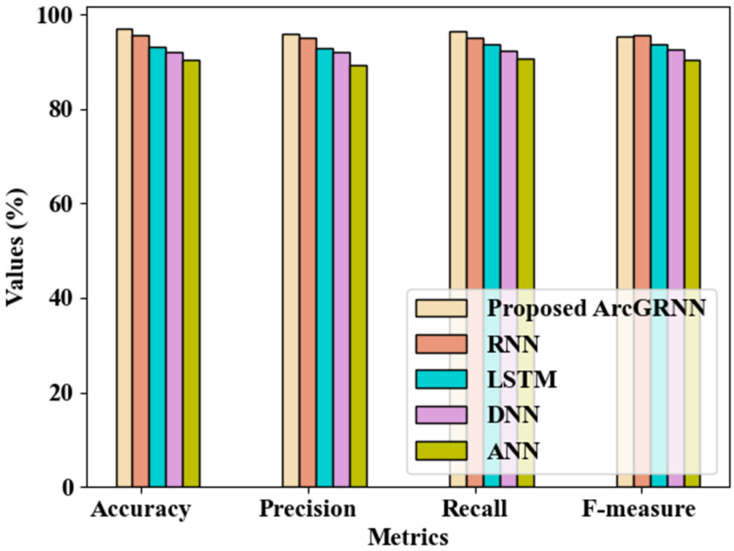
Comparison of the proposed bus bay detection method with existing methods.

Figure 4 illustrates the proposed model’s performance in detecting the desired bus in comparison with the existing models. Regarding its accuracy, precision, recall, and f-measure, the proposed technique’s performance was analyzed in comparison with other techniques including the RNN, Long Short-Term Memory (LSTM), Deep Neural Network (DNN), and Artificial Neural Network (ANN) models. The proposed system detected bus bays with 96.932% accuracy, 95.872% precision, 96.328% recall, and 95.363% f-measure, which were all higher when compared to those of conventional network models. In particular, the average accuracy, precision, recall, and f-measure attained by the prevailing techniques were 92.743%, 92.294%, 92.891%, and 93.016%. The ArcGRNN model effectively detected the bay, as it was detected based on the GPS location and cloud database.

Table 4 details the bus bay detection performance with respect to specificity, sensitivity, and processing time. It can be observed that the proposed approach achieved 95.714% specificity and 96.328% sensitivity, which are higher compared to those of the prevailing networks. In particular, the detection performance of the proposed model was enhanced due to the mitigation of the gradient vanishing problem. Moreover, the proposed model carried out processing within a much shorter time (174,405 ms), when compared to the average time taken by the existing models (505,242 ms). This indicates that the bus bay is efficiently identified by the proposed network.

**Figure 5:**
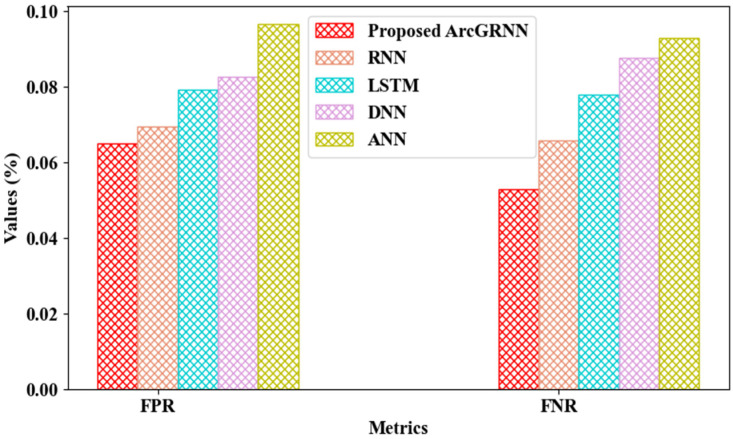
Graphical analysis of FPR and FNR for bus bay detection.

As specified in Figure 5, the bus bay detection performance was evaluated according to the False Positive Rate (FPR) and False Negative Rate (FNR). The ArcGRNN can properly detect the bus bay, as the person’s voice data are pre-processed and converted to text before detection. It can be seen that the proposed technique achieved an FPR and FNR of 0.0652% and 0.0529%, respectively, while the prevailing networks achieved an average FPR and FNR of 0.0820% and 0.0811%, respectively, higher than the proposed technique. This demonstrates that the performance of the proposed technique in terms of bus bay detection was improved, when compared to traditional approaches.

In Table 5, the performance of the proposed method for bus bay detection is detailed, regarding the obtained Positive Predictive Value (PPV) and Negative Predictive Value (NPV). As the speech data are preprocessed for noise removal and converted to text prior to data training, the learning efficiency of the model is improved. Further, tackling the gradient vanishing issue through the use of the Arctan Gradient activation function aids in improving the detection performance, leading to a PPV of 96.74% and an NPV of 95.83%. For comparison, the existing RNN and LSTM attained PPVs of 94.20% and 93.41%, respectively. Furthermore, the existing DNN and ANN attained NPVs of 91.37% and 89.36%, respectively, which are lower than that of the proposed technique. Thus, the detection performance of the proposed method is better than those of the existing methods.

**Figure 6:**
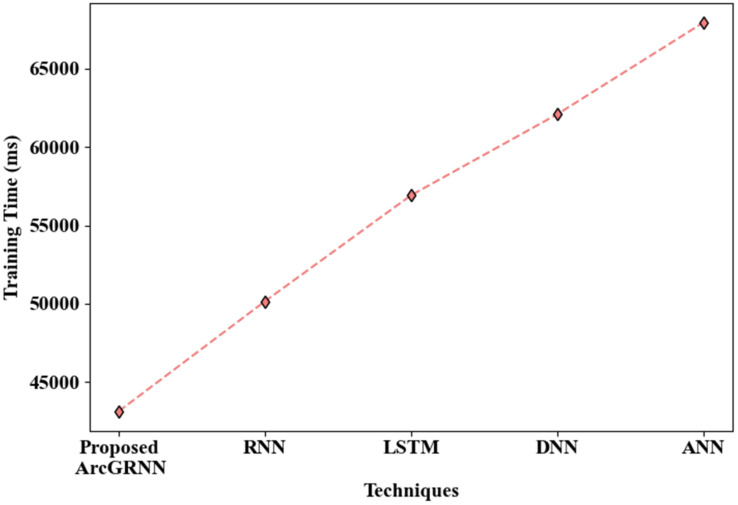
Training time evaluation.

Figure 6 illustrates the performance of the proposed ArcGRNN, in terms of training time, in comparison to the traditional networks. It can be seen that the proposed model consumed a shorter time (of 43,148 ms) for training on the data, due to suppression of the overfitting problem through the use of the Arctan gradient activation function. Meanwhile, the RNN, LSTM, DNN, and ANN models had longer training times of 50,159 ms, 56,921 ms, 62,086 ms, and 67,935 ms, respectively. This is because these existing methods fail to focus on the overfitting or gradient vanishing problem during the backpropagation of data among the neuron layers. Hence, it was also verified that the proposed method is more efficient than the existing techniques.

**Figure 7:**
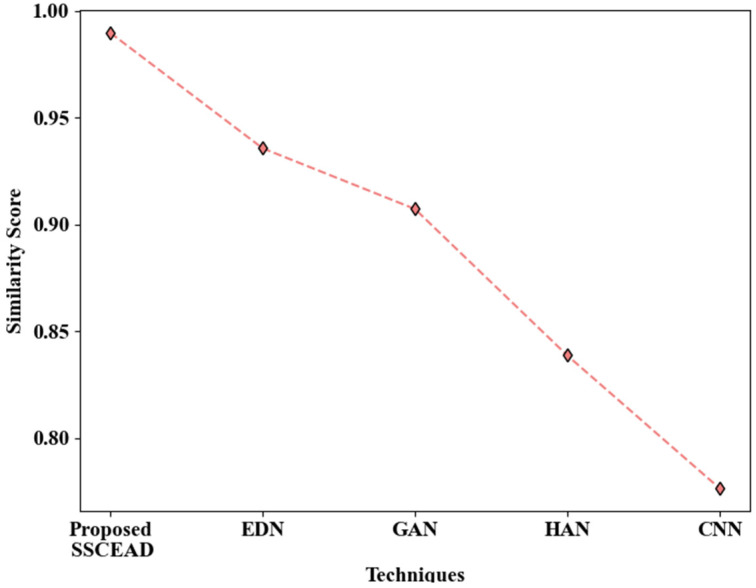
Graphical depiction of similarity scores obtained by text detection approaches.

Figure 7 analyzes the proposed SSCEAD framework’s performance by weighing its similarity score against those of the models used in the comparison. It was found that the proposed technique attained a similarity score of 0.98973, which is higher than that of the prevailing text detection models. In particular, the Encoder–Decoder Network (EDN), General Adversarial Network (GAN), Hierarchical Attention Network (HAN), and CNN achieved similarity scores of 0.93565, 0.90721, 0.83862, and 0.77638, respectively, all lower than that attained by the proposed model. The learning capability of the proposed model was enhanced due to the use of SSC. Thus, the performance of SSCEAD in text detection provides an improvement over the other approaches.

With respect to detection time, the performance of the proposed technique regarding text detection was also evaluated. Table 6 shows that the proposed SSCEAD technique took 160,738 ms to detect text, while the existing techniques took an average of 270,851 ms, longer than that of the proposed approach. The usage of the ArcGRNN model in text decoding helps in the rapid detection of text from images. Hence, when compared to the state-of-the-art approaches, the proposed technique could detect text in less time.

**Figure 8:**
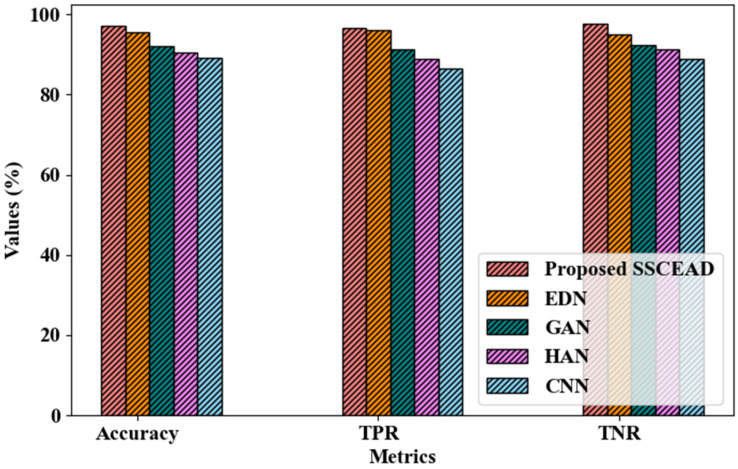
Performance evaluation of the proposed SSCEAD.

Figure 8 analyzes the presented SSCEAD technique for text identification regarding its accuracy, True Positive Rate (TPR), and True Negative Rate (TNR). The proposed model detected text with an accuracy of 97.19%, TPR of 96.55%, and TNR of 97.61%, which are all higher than the metrics obtained with the existing approaches. Among the prevailing techniques, the CNN displayed the lowest performance in text detection, with 89.25% accuracy, 86.56% TPR, and 89.02% TNR. The proposed technique efficiently recognizes text as the receptive field is expanded through pixel shuffling in the convolution kernel.

### 4.3. Comparative Analysis with Related Works

In the framework of Suman et al. [23], who developed a stick using an RNN for VIPs, the obstacles on the path are analyzed for independent navigation. Utilizing this system, obstacles were detected with 91.6% accuracy; however, safer navigation after detecting obstacles was not considered. In this line, Bai et al. [25] presented a wearable travel aid device. To improve the perception of VIPs, this device was made using a CNN to be deployed on eyeglasses and smartphones. However, this device was not sufficient to enable travel from one place to another using a transport system. Hence, based on an improved YOLOv5 network, a bus detection model was proposed by Arifando et al. [22]. This model efficiently assists VIPs in detecting buses with 93.9% precision. Although the bus could be detected by the VIP, it only became efficient once they recognized the bus route name and number. Hence, Sujata Dash Subhendu Kumar Pani [18] detected the bus with the YOLOv3 network and segmented the bus board utilizing a transfer learning technique. Through the LSTM-based Tesseract tool, the route details were converted to text. This technique can detect buses with 90% accuracy and segment the bus board with 80% accuracy. However, to properly guide the VIP, recognizing the route number from the bus is necessary. Using RF and Haar-like filter-based approaches, the bus board area was identified. Next, the route number was recognized through the use of a pattern-matching approach. Tan et al. [16] assessed the bus route number with a detection rate of 56%. Nevertheless, none of these previous works have concentrated on the selection of an appropriate bus bay among the queue of buses in the bus station. Hence, the proposed method selects the optimal bus bay from the bus line utilizing multiple data sources. When identifying the relevant bus, the optimal bay could be selected within 247,891 ms. Moreover, the bus route number was shown to be segmented with 97.19% accuracy. This signifies that the proposed transportation system for VIPs outperforms the state-of-the-art techniques in this area.

#### Comparative Analysis with Similar Works Based on the MS-COCO Dataset

Next, the performance of the proposed system was assessed on the MS-COCO dataset, comparing it with related existing methods.

Table 7 shows a comparative analysis of the proposed system with respect to accuracy, precision, and f-measure. The proposed model exhibited a higher accuracy (96.93%), precision (95.87%), and f-measure (95.36%) for bus bay detection. Meanwhile, the existing ResNet 50 attained 61.50% precision, YOLO version 5 attained 94% f-measure, Neural Architecture Search (NAS) attained 86.30% precision, and Efficient Featurized Image Pyramid Network (EPIFNet) attained 31.60% precision. Further, the ANN attained 83% accuracy and 80% f-measure, which are both lower than those of the proposed system. The superior performance of the proposed method is due to its utilization of the Arctan Gradient Activation Function (Arc-GAF), which improves its learning ability through expanding smaller input data and converging against larger values. In this way, the vanishing or exploding of gradients during the backpropagation is suppressed. Therefore, the performance of the proposed network was found to be enhanced, when compared to the related object detection techniques.

### 4.4. Practical Applicability of the Proposed System

In practice, the voice data of the VIP are collected through a voice assistance module that has an inbuilt microphone. Then, the collected data can be pre-processed using a device that is embedded with the proposed pre-processing approaches. Hence, the redundancy or noise in the voice data is removed, and the resulting clear signal is translated into text format using an installed text converter tool. Further, the retrieved text is sent as a message to the sensor device, which is installed in the wearable aid of the VIP. Then, according to the received text, the GPS location of the VIP and the bay details—namely, the bus bay and optimal bay—are intimated to the VIP. Simultaneously, the sensor captures an image of the destination bus in the bay via GPS. As per the proposed model, the text on the bus is detected, segmented, and extracted, and its similarity with the VIP’s voice data is assessed by the sensor. If the similarity is high, then the sensor transmits a message to the RFID tag, which is fixed in the bus. This informs the bus drivers and conductors about the VIP waiting at the bus station so that they can pick them up accordingly. In the case that the similarity level is very low, the process is iterated from recapturing of the VIP’s voice data. Therefore, the proposed model can be practically applied in the real world to assist VIPs in accurately boarding their desired bus.

### 4.5. Discussions and Limitations

From the simulated outcomes, the bus bay was exactly detected, and the optimal bay was selected with better performance by the proposed model, when compared to existing methods, as the inclusion of the Arctan gradient activation function in the traditional RNN allowed for effective processing of the input data. Thus, the bus bay was detected with higher accuracy, precision, and recall. Furthermore, introducing the EPAS into the proposed algorithm aids in selecting the optimal bay among the bus lines within a short time. The destination bus image is pre-processed, and the text in it is detected and segmented using the proposed SSCEAD, leading to higher accuracy and lower detection time. The learning efficiency of the CNN was also improved by expanding its receptive field using SSC, enabling more effective text detection. Subsequently, the similarity analysis between the extracted text and the pre-processed input voice aided in the decision to inform the bus coordinators. As various data sources and optimal bus bays were taken in to consideration to develop an effective transportation navigation system, the proposed model achieved better performance than those proposed in prevailing works in the related literature.

However, while applying the proposed framework in real-time, environmental factors can affect the transmission of data and may cause delays. Furthermore, the safety of the VIP after reaching and boarding the bus is not considered in this work. These are considered as limitations of this study. We will attempt to rectify these limitations in future work through the use of advanced deep learning techniques. In this regard, various obstacles—including the height of the bus stop from the ground, objects in the step, and environmental data—need to be analyzed to resolve some of the limitations of this study.

## 5. Conclusions

This study proposed an effective system to assist in the navigation of VIPs via bus transport, based on MNC-FR and EPBFTOA. The speech data of the VIP are first subjected to voice pre-processing, followed by text conversion. Then, to identify the desired bus and inform the VIP, optimal bay selection, image pre-processing, bus identification, text detection and segmentation, route similarity analysis, and decision-making processes are carried out. The superior performance of the proposed work was examined through comparing it with state-of-the-art techniques. The desired bus was accurately detected by the VIP using the proposed approach, due to selection of the optimal bay line. The obtained outcomes validated that the proposed model presents improved performance when compared to the existing techniques. Using the proposed technique, the optimal bay was selected within 247,891 ms, and the decision rule was generated within 79,932 ms. Moreover, to access the bus, the bus bay was detected with 96.932% accuracy, and text was identified with 97.19% accuracy. Thus, the proposed approach enables the development of an effective guidance system for blind and Visually Impaired People. 

### Future Scope

Although the bus was detected for the VIP using multiple data sources, we did not concentrate on the person’s safety when boarding the bus. Thus, this work will be extended in the future through determining the obstacles faced by VIPs while boarding the bus.

## Figures and Tables

**Table 1:** Comparative summary of related works.

Related Works	Objective	Methods Used	Advantages	Result	Drawbacks
[16]	Identification of bus route numbers	RF algorithm and pattern matching method	Bus details were accurately captured using the Lucas–Kanade tracker	Achieved a higher detection rate	Features relationships were not recognized by RF, resulting in inaccurate detection.
[17]	Navigation system for the VIP	YOLOv3, Environment-aware Bald Eagle Search, and Actor–Critic algorithm	The person’s query was analyzed, and the obstacles on the path were detected	Higher latency and detection accuracy	Trade-offs occurred when using the Actor–critic algorithm for navigation decisions.
[18]	Wearable device formulation for the safe traveling of VIPs	YOLOv3 and transfer learning method	The detected bus board number is communicated to the person in a voice format	Attained more accuracy	Smaller objects were not detected by the anchor box of YOLOv3.
[19]	Navigation device to assist the blind person	Robot operating system and Fuzzy logic algorithm	Safer directions were alerted to the person	Obtained lower collision	The Fuzzy rules were developed based on assumptions, thereby degrading the decision efficiency.
[20]	Smart glass system for the independent movement of blind persons at night-time	U2-Net and Tesseract model	Path image was expressed in a tactile graph, and the related information was translated into speech format	Detected objects in the path with improved accuracy and precision	Text was not properly analyzed from the low-quality and poorly lit images.
[21]	Mobile application-based navigation assistance for blind persons	CB application and OCR system	The utilization of way-finding footage provided accurate information on the path	The path was identified with higher accuracy, precision, and f1-score	Image data in different formats could not be processed by the OCR.
[22]	Lightweight bus detection network for VIP	Improved YOLO with slim scale detection module	The lightweight approach facilitated real-time detection of the bus	The bus was detected with high accuracy and precision	YOLO involved fewer parameters, thus resulting in sub-optimal detection.
[23]	Vision Navigator framework for the blind and VIP	RNN and single-shot mechanism	Stick and sensor-equipped shoes were utilized for identifying obstacles on the path	Achieved a high accuracy rate	Complex data patterns were not learned by the RNN due to gradient vanishing.
[24]	Mobile application-centered bus stop identification system for VIPs	Neural network-based AAA	The bus stop signs were processed for recognizing bus stops	Distance or deviation between actual location and identified bus stop location was lower	The application required a large number of labeled data and high-quality images for detection.
[25]	Wearable device assistance for the navigation of VIPs	CNN	The device was developed with an in-built camera for better detection of objects	Depicted higher safety and efficiency	Sequential data were not effectively learned by the CNN, which restricted its real-time object detection performance.

**Table 2:** Image outcomes of the proposed technique.

Input Speech Signal	.mp3 Audio File		
**Noise-removed signal**	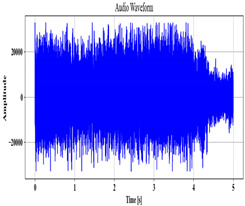	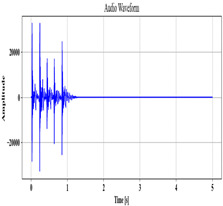	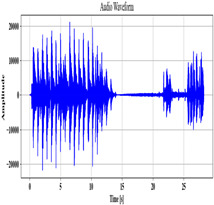
**Speech-to-Text conversion**	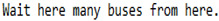	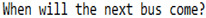	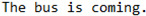
**Input image**	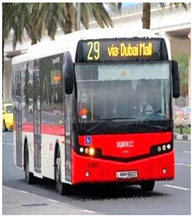	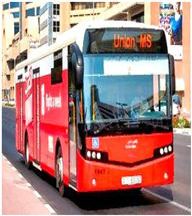	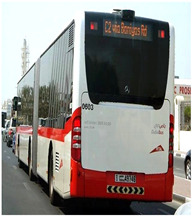
**Noise-removed image**	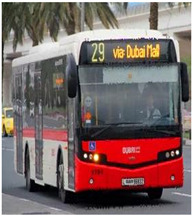	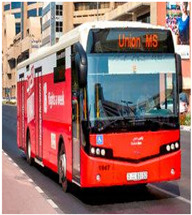	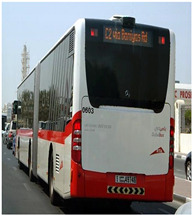
**Contrast-enhanced image**	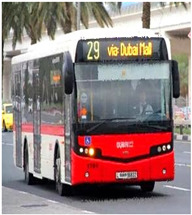	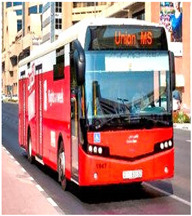	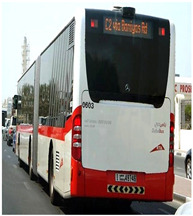
**Identified Bus**	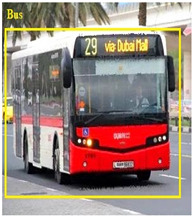	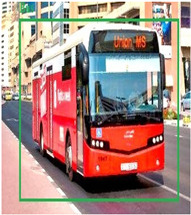	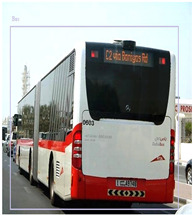
**Text identification**	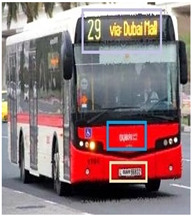	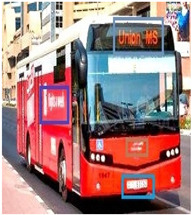	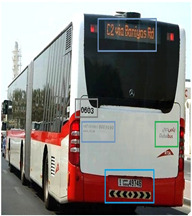
**Text extraction**	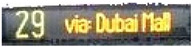	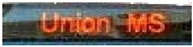	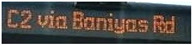

**Table 3:** Performance analysis of the proposed optimal bay selection method.

Methods	Optimal Bay Selection Time (ms)	Average Fitness
Proposed EPBFTOA	247,891	9.068
BFOA	389,124	19.8787
MRFO	596,757	42.0519
AVOA	804,628	67.5498
BESO	997,245	79.8054

**Table 4:** Performance of bus bay detection.

Techniques	Specificity (%)	Sensitivity (%)	Processing Time (ms)
Proposed ArcGRNN	95.714	96.328	174,405
RNN	94.285	95.019	407,395
LSTM	93.09	93.652	428,012
DNN	91.788	92.301	562,513
ANN	90.394	90.595	623,048

**Table 5:** Bus bay detection analysis.

Methods	PPV (%)	NPV (%)
Proposed ArcGRNN	96.74	95.83
RNN	94.20	94.12
LSTM	93.41	92.35
DNN	92.08	91.37
ANN	90.16	89.36

**Table 6:** Performance comparison of text detection.

Techniques	Detection Time (ms)
Proposed SSCEAD	160,738
EDN	286,059
GAN	215,191
HAN	260,425
CNN	321,728

**Table 7:** Performance comparison on the MS-COCO dataset.

Authors	Technique Used	Precision (%)	Accuracy (%)	F-Measure
Proposed	ArcGRNN	95.87	96.93	95.36
[26]	ResNet 50	61.5	-	-
[27]	YOLO version 5	-	93.00	94.00
[28]	NAS	86.30	-	-
[29]	EFIPNet	31.60	-	-
[30]	ANN	-	83.00	80.00

## Data Availability

Data are contained within the article.
